# The Malay version of the attitudes and beliefs about cardiovascular disease (ABCD-*M*) risk questionnaire: a translation, reliability and validation study

**DOI:** 10.1186/s12889-022-13811-8

**Published:** 2022-07-25

**Authors:** Zarudin Mat Said, Tengku Alina Tengku Ismail, Anees Abdul Hamid, Ramesh Sahathevan, Zariah Abdul Aziz, Kamarul Imran Musa

**Affiliations:** 1 Disease Control Unit, Hilir Perak District Health Office, Jalan Maharajalela, 36000 Teluk Intan, Perak Malaysia; 2 Department of Community Medicine, School of Medical Sciences, Health Campus, Universiti Sains Malaysia, Health Campus, 16150 Kubang Kerian, Kelantan Malaysia; 3 Primary Care Unit, Kelantan State Health Department, Tingkat 5, Wisma Persekutuan, Jalan Bayam, 15590 Kota Bharu, Kelantan Malaysia; 4 Department of Medicine and Neurology, Ballarat Health Services, Drummond Street North, Ballarat, Victoria 3350 Australia; 5 Department of Medicine, Hospital Sultanah Nur Zahirah, 20400 Kuala Terengganu, Terengganu Malaysia

**Keywords:** Stroke, Cardiovascular, Awareness, Reliability, Validity, Malay

## Abstract

**Background:**

Cardiovascular disease (CVD) and stroke are global public health problems and cause high mortality, especially in low- and middle-income countries. Knowledge and awareness are critical points in managing the risk in the general population. The Attitudes and Beliefs about Cardiovascular Disease (ABCD) risk questionnaire was developed to evaluate the awareness of stroke and CVD risk. Thus, the government can set up a practical risk assessment and management programme. The initiative will encourage people to seek healthcare timely and reduce the possibilities of developing complications.

**Objective:**

This study aimed to translate and validate the ABCD risk questionnaire into the Malay language and evaluate the psychometric properties of the Malay version in the general population in Malaysia.

**Methods:**

The questionnaire was translated using a standard forward–backwards translation method. The validation was perfomed by both expert panels and a potential user group. Next, the exploratory factor analysis was conducted to examine factorial validity. The respondents were selected from the government health clinics and according to the study criteria irrespective of the CVD risk. We used Cronbach’s alpha and Raykov’s rho to explore the internal consistency and composite reliability of the 18 items from three domains. Finally, the confirmatory factor analysis (CFA) was conducted using a robust maximum likelihood estimator.

**Results:**

The content and face validity indices were determined to be 0.94 and 0.99 respectively. Data were obtained from 179 respondents (mean age, 36.8 years; female, 68.2%; secondary level education, 51.1%). The internal consistency and composite reliability of the domains showed good results ranging from 0.643 to 0.885. The factor loadings of each item were acceptable (> 0.3), and the fit indices from the CFA resulted in a good model fit [χ^2^ (*p-*value = 0.16), SRMR = 0.054, RMSEA = 0.029, CFI = 0.99, TLI = 0.99)].

**Conclusions:**

The Malay version of the ABCD risk questionnaire is a valid and reliable tool to assess the awareness of stroke and CVD risk in the general population in Malaysia.

**Supplementary Information:**

The online version contains supplementary material available at 10.1186/s12889-022-13811-8.

## Introduction

Cardiovascular disease (CVD) is a global public health problem and causes high mortality, especially in low- and middle-income countries [1, 2]. The World Health Organisation estimated that 17.9 million people die annually from CVDs mainly due to coronary artery disease and stroke, which accounted for 32% of global deaths [3]. In Malaysia, the leading cause of CVD death in 2020 was coronary artery disease (15.0%), followed by stroke (8.0%) [4]. In addition, stroke has been considered as the most common cause of adult disability severely affecting the quality of life [5–7]. Therefore, the need for prevention has become a significant challenge. A personalized risk assessment of the risk factors for stroke and CVD is significant for developing prevention strategies, particularly in the young- and middle-aged populations [8]. A proper stroke risk assessment method including the evaluation of knowledge and awareness and lifestyle modification (i.e. smoking cessation, physical activity, healthy diet and maintaining body mass index, low cholesterol levels and normal blood pressure and fasting plasma glucose levels) is vital to reduce the incidence of not only stroke and CVDs but also other non-communicable diseases (NCDs) (i.e. chronic kidney disease, cognitive impairment and diabetes) [8, 9]. It is expected that an increased knowledge and awareness of these conditions will prompt people to seek healthcare timely and reduce the possibility of developing complications.

There is a need to assess the level of knowledge and awareness of stroke and CVD risk in the general population, perception of the risk and readiness to change behaviours. One way of measuring them is through a valid and reliable questionnaire. To the best of our knowledge, most of the questionnaires only assessed an area of the stroke risk, either knowledge or perception. Furthermore, the questionnaire’s targeted population mainly refers to the person with a history of stroke or relatively related to patients with stroke. Several questionnaires were developed to measure knowledge, perceptions of CVD and intention to change behaviours, but most of them were non-validated, lengthy, and non-specific [10–12]. The use of non-validated tools may produce questions that are inaccurate and reliably capture individuals’ views or measure what they intend to measure [13].

The Attitudes and Beliefs about Cardiovascular Disease (ABCD) risk questionnaire may evaluate the accuracy of the perceived CVD risk, general knowledge of CVD and intention to change behaviour in regard to diet and exercise in the general population. It is a new set of questionnaire that was developed to evaluate the awareness of stroke and CVD risk among the National Health Service (NHS) Health Check attendees recently [13]. The development was guided by the Health Belief Model (HBM) and Transtheoretical Model (TTM) [14, 15]. The individuals who have accurate knowledge of stroke and CVD, perceive susceptibility to and consequences of the disease and are aware of the benefits of taking preventive measures are more likely to make significant lifestyle choices to prevent the onset of disease [14].

The questionnaire was tested in England’s NHS Health Check programme (i.e. stroke and CVD prevention programme) as there was no instrument measuring stroke and CVD risk awareness before [13]. Therefore, the questionnaire is relevant to assess the knowledge of stroke and CVD, perception towards the benefits and risks and intention to change in the broader scope of the population. Moreover, the original version was developed on a non-risk-stratified population representing the general population; even some of the results may limit the representativeness. However, it is significant to note that the questionnaire showed satisfactory reliability and validity and was brief and easy to use.

However, the valid ABCD questionnaire was not available in the Malay language. Moreover, different languages, cultures and populations may make it difficult to accurately capture the local target population’s thoughts, feelings, perceptions, behaviours and attitudes.

Therefore, the current study aimed to translate the ABCD risk questionnaire to the Malay language and evaluate the psychometric properties of the Malay-translated version within a sample of the general population, especially among young adults, to assess its cross-cultural validity. The valid and reliable Malay version of the ABCD risk questionnaire will be useful to measure the stroke and CVD risk awareness in the Malay-speaking population, especially in countries such Malaysia, Indonesia, Brunei and Singapore.

## Methods

### Study settings and participants

A cross-sectional study was conducted in Kelantan, Malaysia, involving four districts: Bachok, Machang, Tanah Merah and Pasir Puteh. The respondents were adults (aged ≥18 years) who attended the government health clinics during the study periods. The health clinics that serve the most patients in every selected district were included in this study. The respondents who came to the chosen health clinics on recruitment days with no cognitive impairment (as judged by an attending researcher) and had good Malay language command were then invited to participate in the study. The chosen respondents were not restricted to those who are free of CVD risk or any stage of CVD risk. Data collection was performed between January 2020 to March 2020. The sample size was determined for the reliability testing and factor analysis, which required at least 164 respondents for a scale with ≤4 factors with the expected factor loading (FL) of ≥0.50 and item communality of < 0.45 [16, 17]. As regards the possible dropout, we oversampled the size by 10%; hence, the minimum required sample size was 173 respondents.

However, we recruited 179 randomly selected respondents based on the attendee list from the chosen health clinics. The study protocol and rights of the participants were explained. Those who consented to participate in the study signed informed consent forms and were given questionnaire forms to be completed.

### Instrument

The self-administered questionnaire consists of 26 items constituting four scales or domains: (a) knowledge of stroke risk and prevention, eight items; (b) perceived risk of heart attack/stroke, eight items; (c) perceived benefits and intentions to change, seven items; and (d) healthy eating intentions, three items. For the ‘knowledge of stroke risk and prevention’ domain, the type of answer is true/false/do not know, while the rest are according to the 4-point Likert scale: 1, strongly disagree; 2, disagree; 3, agree; 4, strongly agree; and 0, non-applicable. For the English version, the average time taken to complete the questionnaire (26 items) by each participant was between 10 and 15 min. The overall score was calculated from all item scores by the domains. The higher the score, the higher the awareness and the readiness to change the behaviour.

### Translation and adaptation process

The ABCD risk questionnaire was translated into Malay using international guidelines for cross-cultural adaptation [18–20] to ensure the quality of the translated version and its consistency of meaning to the original version [21]. There were four translators in this study (two performed the forward translation, and another two the backward translation). First, the forward translation process (from English to Malay) was conducted by two independent translators, and the translators produced a report of the translation. After a thorough discussion, the two translations were synthesised into one document by ZMS, who also addressed any gaps or differences between the two reports.

Then, the original and translated versions of the questionnaire were given to another two different translators. Both were native Malay speakers who spoke English as their second language. The translators who received either the original or translated questionnaire version then performed the forward or backward translation, respectively. Finally, the forward and backward translation discrepancies were reconciled, and cross-cultural adaptation was made to derive the final version. As the primary purpose of the ABCD risk questionnaire is to act as a tool to evaluate the awareness of stroke and CVD risk in the general population, the adapted questionnaire in the Malay version is called *Soal Selidik Kesedaran mengenai Penyakit Kardiovaskular* (ABCD-*M*).

### Validation process

The translated questionnaire subsequently underwent content validity, face validity, construct validity and reliability (internal consistency and composite) assessment. Content validation aims to assess the relevancy and representativeness of each item to a specific domain by a panel of experts. This context will evaluate the relevance of all 26 items in the questionnaire to represent each domain; ‘knowledge of stroke risk and prevention’, ‘perceived risk of heart attack/stroke’, ‘perceived benefits and intentions to change’ and ‘healthy eating intentions’.

The content validation of the questionnaire was conducted by five experts (a public health physician, neurologist, family health physician, statistician, and general practitioner). They were asked to give a score from 1, item not relevant, to 4, item very relevant, based on the relevancy of the translated items in the questionnaire. The scores of 3 and 4 were recategorised as 1 (relevant), and the scores of 1 and 2 were recategorised as 0 (not relevant).

Face validation testing, which aims to assess the clarity and comprehensibility of the translated items, was conducted by 10 potential target users in the adult population [22]. The users were asked to give a score from 1, item not clear and not understandable, to 4, item very clear and understandable, based on the clarity and comprehensibility of the translated items in the questionnaire. The scores of 3 and 4 were recategorised as 1 (clear and understandable), and the scores of 1 and 2 were recategorised as 0 (not clear and understandable).

Then, the construct validity of the translated questionnaire was evaluated by estimating its association with other variables (or measures of a construct) with which it should be correlated positively, negatively or not at all [23]. Theoretically, construct validity is the degree to which an instrument measures the trait or theoretical construct that it is intended to measure [23, 24]. It is the most valuable and most challenging measure of validity. The purpose of construct validity can be obtained using the factor analysis [24, 25]. It is usually employed when the construct of interest is in several dimensions, forming different domains of a general attribute [25]. In the analysis, several items put up to measure a particular extent within a construct of interest are supposed to be highly related to one another than those measuring other dimensions. An exploratory factor analysis (EFA) was conducted utilising principal component analysis with the varimax rotation method [26]. Items loaded above 0.30 and non-problematic cross-loading were considered for further analysis. The problem is indicated by having almost comparable FLs in two or more factors, indicating that the item is not specific for a construct and general [17, 26]. Therefore, the factor analysis results will satisfy the criteria of construct validity, including both the discriminant validity (loading of at least 0.40, no cross-loading of items above 0.40) and convergent validity (eigenvalues of 1, FL of at least 0.30, items that load on posited constructs) [25, 26].

### Reliability test

The reliability was further tested using an internal consistency measure, the Cronbach’s alpha coefficient. The test is viewed as the most appropriate measure of reliability when using Likert scales [26]. Most of the literature agreed on a minimum internal consistency coefficient of 0.70 even though no absolute rules exist for internal consistencies [26–28].

The suggested four cut-off points for reliability included excellent reliability (0.90 and above), high reliability (0.70–0.90), moderate reliability (0.50–0.70) and low reliability (0.50 and below) [26, 29].

**Fig. 1:**
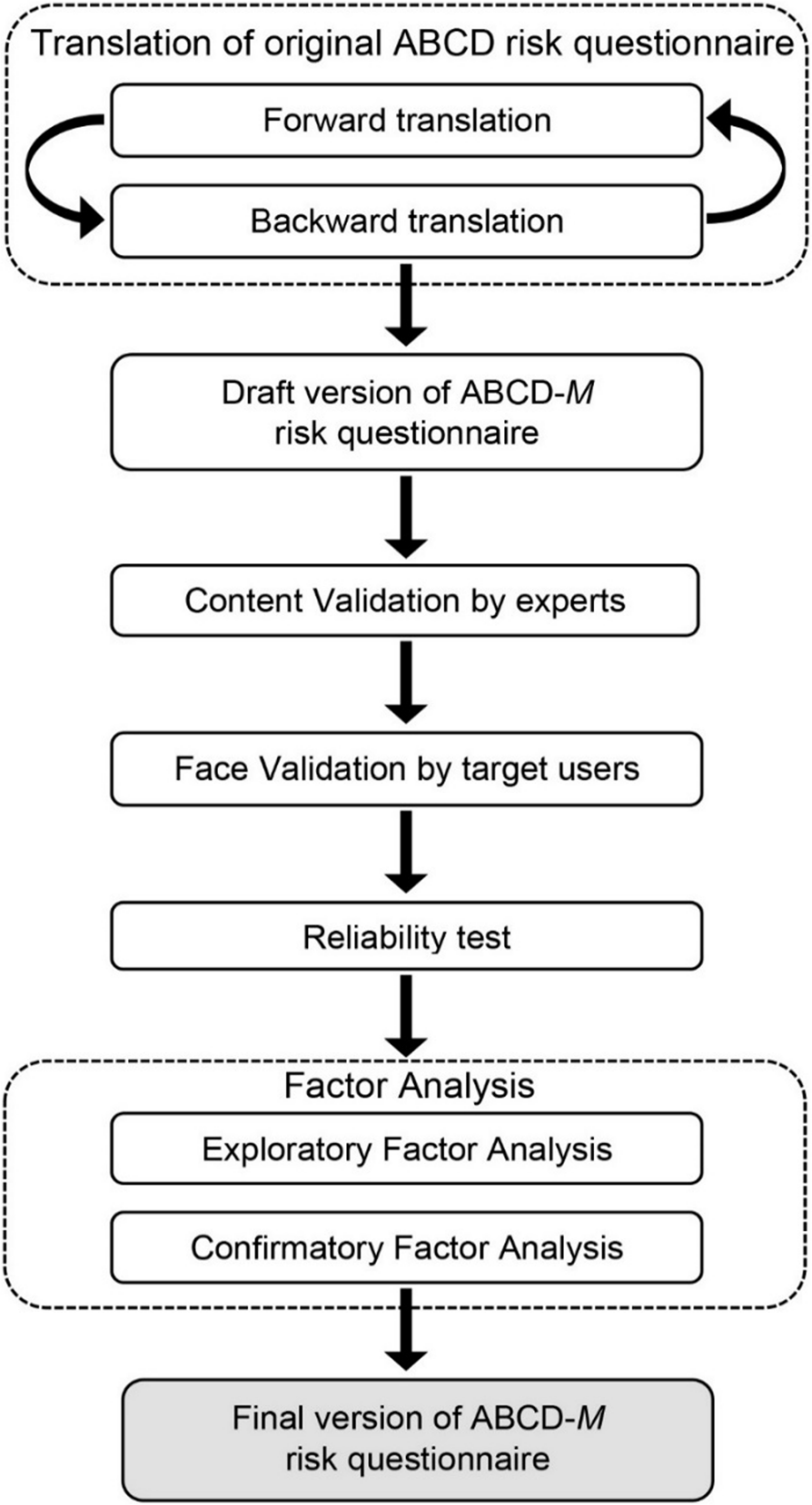
Flowchart of the translation and validation of the Attitudes and Beliefs about Cardiovascular Disease risk questionnaire

Next, the composite reliability (Raykov’s rho) value was estimated using the confirmatory factor analysis (CFA). It is more general in that it does not assume that the items are unidimensional and considers the correlated errors [17, 30]. Raykov’s rho values of 0.7 and above were considered as good reliability [31, 32]. The flow of translation, validation process and reliability testing are depicted in Fig. 1.

### Data analysis

For the validation test, the content validity index (CVI) and face validity index (FVI) were computed by calculating the scale average [22, 33]. The item-level CVI (I-CVI) was the proportion of content experts giving the item a relevance rating of 3 or 4, which is calculated by dividing the agreed item by the number of experts in the study. At the same time, the CVI average was estimated by dividing the sum of the I-CVI scores by the number of items. Next, the item-level face validity index (I-FVI) was defined as the proportion of respondents giving an item a clarity and comprehension rating of 3 or 4. By measuring the average of the I-FVI scores judged by all respondents, the FVI of the questionnaire was estimated. The acceptable CVI and FVI values were at least 0.83, corresponding to the number of experts and respondents involved in this study [22, 34].

The baseline characteristics of the respondents were presented according to the type of variables: frequency (n), percentage (%) and mean ± standard deviation (SD). The factor analysis was further conducted – a multivariate statistical analysis to determine the FLs, correlations among the items, internal consistency and composite reliability. The analyses were primarily performed with RStudio IDE [35] using the *lavaan* and *semTools* packages [36, 37]. All 18 items from three domains, ‘perceived risk of heart attack/stroke’, ‘perceived benefits and intentions to change’ and ‘healthy eating intentions’, were analysed using the factor analysis. In contrast, the eight remaining items under the ‘knowledge of stroke risk and prevention’ domain could not be entered into the factor analysis because of the different scales used [13]. In the EFA, the items with FLs above 0.30 were considered acceptable [17]. Multicollinearity between the factors was identified when the factor-to-factor correlation was above 0.85 [38]. Internal consistency reliability and composite reliability were estimated, and values ≥0.7 were considered to reflect good reliability [29, 32].

A robust maximum likelihood estimator was used for the CFA as we assumed that data were not normally distributed data [38]. The model fit assessment was based on the fit indices with the respective cut-off values: chi-square (*χ*^2^) (*p-value* > 0.05), comparative fit index (CFI) and Tucker–Lewis fit index (TLI) of ≥0.95 (good) or ≥ 0.90 (acceptable), root mean square error of approximation (RMSEA) of ≤0.08 and standardised root mean square residual (SRMR) of ≤0.08 [38]. A model-to-model comparison was based on the Akaike information criterion (AIC) and Bayesian information criterion (BIC). The model with the lowest values of the AIC and BIC was the best fitting model for the CFA [31, 39].

### Ethical consideration

This study was approved by the Medical Review and Ethical Committee (MREC) of Ministry of Health (MOH) Malaysia (NMRR-19-3296-51,864-IIR), Human Research Ethics Committee Universiti Sains Malaysia (USM), Malaysia (USM/JEPeM/19110815); and Kelantan State Health Department. Data confidentiality was maintained, and only the researchers had access to the data. The permission to conduct translation and adaptation of the ABCD risk questionnaire was obtained from the original authors.

## Results

The review of the forward–backward translation addressed several improvements to enhance the accuracy of the Malay-translated version as the original version without compromising the validity and reliability. Throughout the discussion and review with expert panels and a potential user group, all items (*n* = 26) in the four domains were retained as they were deemed important and appropriate. Several words and phrases including *strok* for stroke, *stres* for stress, *aktiviti sederhana berat* for moderately intense activity, *alkohol* for alcohol and *kolesterol buruk* for bad cholesterol were selected after considering the usage of the words and phrases in the Malaysian scenario to define the correct meaning.

Moreover, the domains ‘perceived benefits and intentions to change’ and ‘healthy eating intentions’ have been revised to simplify the sequence of the questions, thereby making them more comprehensible and balanced. The three items from the domain ‘perceived benefits and intentions to change’, items 17, 18 and 21, were joined together with the three items in the domain ‘healthy eating intentions’. As a result, the third domain is renamed as ‘perceived benefits’ with four items, and the last domain is the ‘intention to change’ with six items. The details of the translation are attached in the supplementary material.

The CVI and FVI of the ABCD-*M* risk questionnaire were 0.94 and 0.99, respectively (refer to supplementary material: Tables 1 and 2). Both parameters indicate that all items in the questionnaire are relevant to the domain, clear and understandable for the intended study population. The construct validity and reliability testings were conducted using 179 samples from the targeted population who responded to the adapted and translated questionnaire. The age of the respondents ranged from 18 to 66 years, with a mean of 36.82 (SD, 12.17). Most of the respondents were married (*n* = 133, 75.14%), followed by the statuses single (*n* = 39, 22.03%) and divorced (*n* = 5, 2.82%). The respondents were predominantly female (*n* = 122, 68.16%). More than half of the respondents attended up to the secondary level of education (*n* = 91, 51.12%), followed by the tertiary (*n* = 82, 46.07%) and primary (n = 5, 2.81%) levels of education. The highest number of respondents worked for the government (*n* = 56, 31.28%), followed by self-employed (*n* = 39, 21.79%), housewife (*n* = 31, 17.32%), working in a private sector (*n* = 25, 13.97%), student (*n* = 16, 8.94%) and unemployed (*n* = 12, 6.7%). The distribution of the monthly income showed that 50.31% (*n* = 81) of the respondents earned RM1001 to RM3000 per month, 25.47% (*n* = 41) earned less than RM1000 per month, 13.66% (*n* = 22) earned RM3001 to RM5000 per month and 10.56% (*n* = 17) more than RM5000.

The overall result of the ABCD-*M* revealed that the range was between 6 and 77 (a total of 80 marks) with a mean of 49.26 (61.58%). All domains recorded more than 50% of the full marks, except for the ‘perceived risk of heart attack/stroke’. The domain exhibited a mean of 14.97 (SD, 6.01) (46.78% of the total marks). The sociodemographic characteristics of the respondents are listed in Table 1.

**Table 1:** Sociodemographic characteristics of the respondents

Variables	n (%)	Mean (SD)	Range
**Age**	–	36.82 (12.17)	18–66
**Gender**			
Male	57 (31.84)	–	–
Female	122 (68.16)		
**Status**			
Single	39 (22.03)	–	–
Married	133 (75.14)		
Divorced	5 (2.82)		
**Education**			
Primary	5 (2.81)	–	–
Secondary	91 (51.12)		
College/Uni	82 (46.07)		
**Occupation**			
Unemployed	12 (6.70)	–	–
Student	16 (8.94)		
Housewife	31 (17.32)		
Self-employed	39 (21.79)		
Government	56 (31.28)		
Private	25 (13.97)		
**Income**			
< RM1000	41 (25.47)	–	–
RM1001 – RM3000	81 (50.31)		
RM3001 – RM5000	22 (13.66)		
> RM5000	17 (10.56)		
**Awareness (80 marks)**		49.26 (9.31)	6–77
Knowledge (8 marks)		5.82 (1.58)	–
Perceived Risk (32 marks)		14.97 (6.01)	–
Perceived Benefits (16 marks)		12.26 (2.86)	–
Intention to Change (24 marks)		16.21 (3.43)	–

The EFA iteration confirmed the FLs and reliabilities as reported in Table 2. Most of the items had good FLs (> 0.50) and low complexity, except for item 15 under the domain ‘perceived risk of heart attack/stroke’, which had low FL (0.26) and communality (0.122) and high complexity (2.06). In addition, almost all items had good communality values ranging from 0.32 to 0.94 [17, 40].

**Table 2:** Factor analysis, internal consistency and composite reliability

Domain	Item	Factor Loading	Communality	Cronbach’s Alpha	Raykov’s Rho
**Perceived Risk**	9	0.747	0.589	0.876	0.885
	10	0.882	0.774		
	11	0.939	0.860		
	12	0.822	0.670		
	13	0.761	0.600		
	14	0.690	0.510		
	15	0.263	0.122		
	16	0.539	0.320		
**Perceived Benefits**	17	0.710	0.512	0.806	0.766
	18	0.666	0.465		
	19	0.659	0.439		
	20	0.668	0.449		
**Intention to Change**	21	0.777	0.609	0.696	0.643
	22	0.722	0.533		
	23	0.970	0.940		
	24	0.738	0.544		
	25	0.728	0.529		
	26	0.892	0.797		
**Correlation:** Perceived Risk ↔ Perceived Benefits *r* = 0.141 Perceived Risk ↔ Intention to Change *r* = 0.107 Perceived Benefits ↔ Intention to Change *r* = 0.045					

The internal consistency reliability of the structure was measured using Cronbach’s alpha. The α values of the domains ‘perceived risk of heart attack/stroke’ (eight items), ‘perceived benefits’ (four items) and ‘intention to change’ (six items) were 0.876, 0.806 and 0.696, respectively. Thus, all domains were above the minimum threshold of reliability of 0.70 [29].

**Fig. 2:**
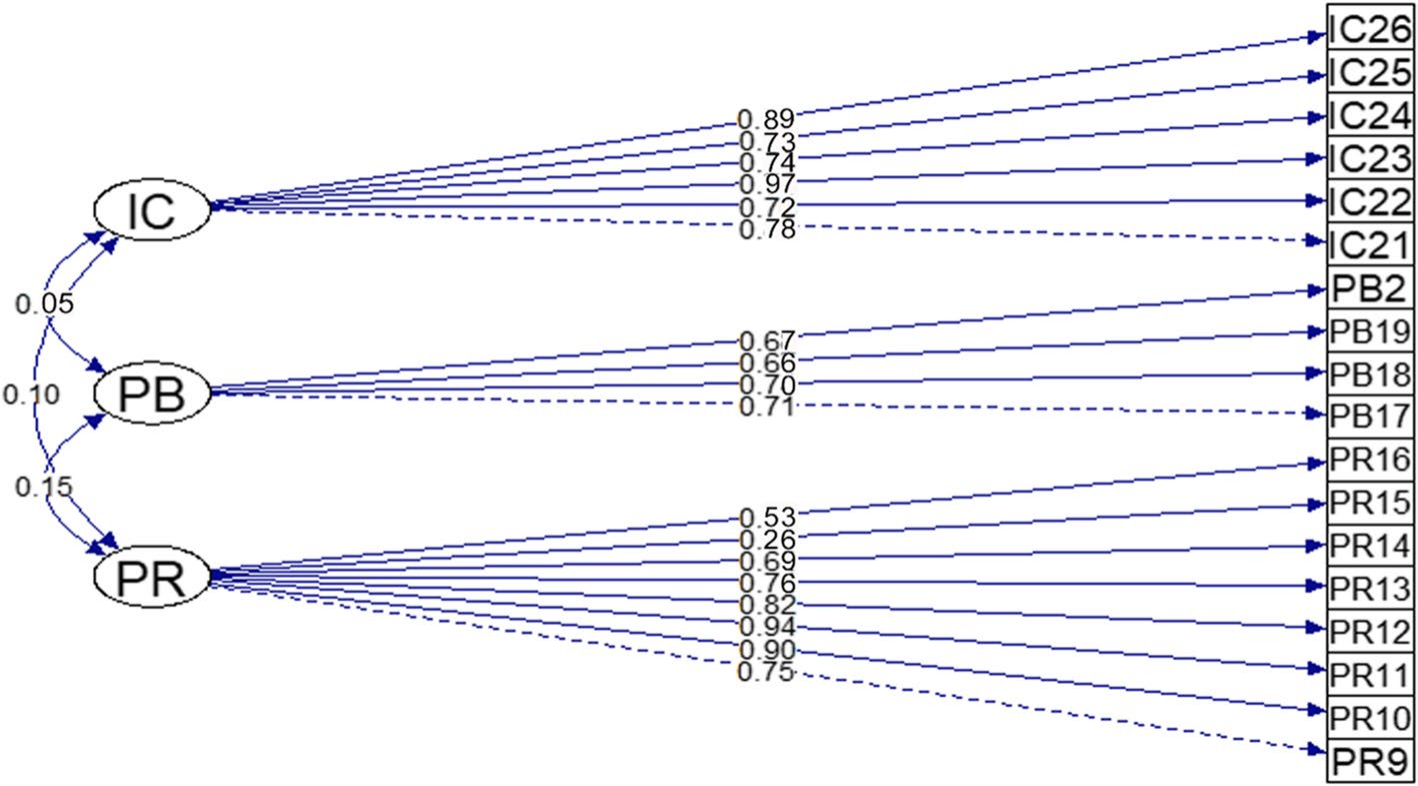
The path diagram of the final model (model 1)

On the other hand, Raykov’s rho of the CFA for each domain was good [32, 41], which ranged from 0.643 to 0.885. The α values of the domains ‘perceived risk of heart attack/stroke’, ‘perceived benefits’ and ‘intention to change’ were 0.885, 0.766 and 0.643, respectively. The correlations between the domains were < 0.85, which indicated the absence of multicollinearity between the items [38]. Hence, the parallel analysis showed that three domains as used in the original questionnaire would be retained. The FLs of each item and correlations between the domains are illustrated in Fig. 2.

The fit indices from the CFA resulted in good goodness of fit (SRMR, 0.054; RMSEA, 0.029; CFI, 0.99; TLI, 0.99) (Table 3). The SRMR and RMSEA values were clearly below the cut-off value of 0.08, while the support from good CFI and TLI values were more than 0.95. The *p-*value for the chi-square statistic (*χ*^2^ [98] = 119.81) was not significant (*p-*value = 0.16), indicating the good model fit. The translated version (model 1) was compared with the modified version (model 2) with the value of the chi-square (*χ*^2^ [9] = 10.37, *p-*value = 0.32). Model 2 consisted of 17 items because of the elimination of item 15 under the domain ‘perceived risk of heart attack/stroke’. Both were fitted well models, with model 2 having a slightly lower AIC of 6307.1 and BIC of 6511.1 compared with model 1.

**Table 3:** Fit indices of the models

Model	χ^**2**^ (df)	***p***-value	SRMR	RMSEA	90% CI	CFI	TLI	AIC	BIC
1	119.81 (98)	0.16	0.054	0.029	0, 0.052	0.99	0.99	6851.8	7084.5
2	101.30 (89)	0.18	0.050	0.029	0, 0.053	0.99	0.99	6307.1	6511.1

Model 1: Original version of 18 items constitutes of domains; ‘Perceived Risk of Heart Attack/Stroke,’ ‘Perceived Benefits,’ and ‘Intention to Change’

Model 2: Modified version of 17 items with the elimination of item-15th under the ‘Perceived Risk of Heart Attack/Stroke’ domain

Model-to-model comparison: χ^2^ diff. (df) = 10.37 (9), *p*-value = 0.32

*SRMR* standardized root mean square residual, *RMSEA* root mean square error of approximation, *CI* confidence interval, *CFI* comparative fit index, *TLI* Tucker-Lewis fit index, *AIC* Akaike information criterion, *BIC* Bayesian information criterion

## Discussions

The validated questionnaire assessing the stroke and CVD risk awareness in the general population is still new in Malaysia. Not only currently available questionnaires focus on the population related to known patients with stroke, but also the area of assessment is often concentrating mainly on the knowledge regarding stroke and CVD [12, 42–45]. Even fewer of the available questionnaires have the concise version with acceptable validity and reliability. The assessment of the stroke and CVD risk awareness in the general population is essential for evaluating the general knowledge of stroke and CVD, accuracy of perception towards the risk of the diseases and benefits of healthy lifestyles concerning diet, exercise, and other related CVD risks. Indirectly, the results of the assessment survey will provide some clues to the government in the way of new policies related to the NCD prevention and its improvement.

The ABCD questionnaire was developed based on a theory-based health promotion model, specifically the HBM. The use of a theory-based health promotion model is crucial in developing or/and translating valid and reliable questionnaires. In addition to ensuring the reproducibility of the new version of the questionnaire and leveraging the true potential of the questionnaire, the intended aim can be captured clearly from the targeted users [46–48]. The HBM and TTM are considered popular models that have predicted changes in knowledge, attitudes and/or behaviours across health behaviours that have varied widely. According to the HBM, people will be more motivated to engage in healthy behaviours if they believe they are susceptible to a specific negative health outcome. Furthermore, the stronger a person’s perception of the severity of the negative health outcome, the greater the motivation to avoid it.

The process of translation and validation of the questionnaire was performed by both expert panels and a potential user group. The review was crucial in identifying mistakes and correcting them so that the content, concept, criterion and semantic criteria matched and were equivalent to the original English version. It ought to be relatively easy to understand for the general population in Malaysia regardless of sociodemographic differences. Furthermore, the use of the EFA and CFA provides more robust evidence to support the validity of the factor structure of a measure [40]. There are limited studies that use the CFA even though the method is proven superior to the EFA and other simple reliability analyses in several respects [31, 49]. However, the CFA is considered an indispensable tool for validation and requires specialised software [31]. This study used *lavaan* and *semTools* packages of the R software, while the EFA can be efficiently conducted in much statistical software. The method could have hindered the use of the CFA in the validation of the original questionnaire.

The internal consistency reliability of the domains was good and improved, with almost similar values to the original questionnaire [13]. In addition, composite reliability from the CFA showed good results even though the values were lower than the EFA, which ranged from 0.696 to 0.876 (by Cronbach’s alpha). It can be attributed to the correlated error covariances in the reliability calculation [31, 50]. Compared with Cronbach’s alpha, Raykov’s rho considers the FLs of every item, leading to higher estimates of true reliability [50]. If the items measured the same single construct and have the same FLs and there were no error covariances, Raykov’s rho and Cronbach’s alpha would be the same, or the discrepancy between the values would be very close [50]. The FLs of the items were good as the values were more than 0.5 [17, 40]. However, item 15 under the domain ‘perceived risk of heart attack/stroke’ had low FL (< 0.30) and communality (< 0.25) and high complexity. The possible reason is that some items had been reverse coded, including items 15 (*Saya* *tidak risau* *bahawa saya mungkin mengalami serangan jantung atau strok*) (I am not worried that I might have a heart attack or stroke), 23 (*Saya* *tidak memikirkan* *untuk bersenam selama 2 ½ jam seminggu*) (I am not thinking about exercising for 2 ½ h a week) and 26 (*Saya* *tidak memikirkan* *untuk makan sekurang-kurangnya lima hidangan buah-buahan dan sayur-sayuran setiap hari*) (I am not thinking about eating at least five portions of fruit and vegetables a day).

As stated in some literature, the reverse coded item is critical to control for and/or identify acquiescence response bias of the respondents [51] and alert inattentive respondents that item content varies [52]. The inclusion of low FL items did not interrupt the overall reliability value of the domain, relevancy and clarity. The alternative model (model 2) with the deletion of item 15 was tested and compared with the current model (model 1). It showed indistinguishable fit indices and insignificant differences from the current model. The decision to accept the model fit was based on the fit indices (robust), which were χ^2^ (*p-*value > 0.05), CFI and TLI of ≥0.95 and RMSEA and SRMR of ≤0.08. As a result, the items used in the original questionnaire were retained, considering the importance and relevancy of every item and domain.

## Limitation of the study

The development of the ABCD risk questionnaire did not encompass all aspects of CVD risk observed in the general population. As explained in the original version, questions on smoking and drinking were omitted as they did not apply to most study participants [13]. At the same time, the evaluation of smoking and drinking risk factors among patients with stroke in Malaysia grossly accounted for 22.4 and 3%, respectively [53]. The inclusion of both risks will improve the quality of the questionnaire and generalisability, specifically in the adult population.

Nevertheless, the current questionnaire has included the area of modifiable risk factors, including hypertension and diabetes, physical activity, healthy and balanced diet intake and alcohol consumption, which are considered as a higher percentage of population-attributable risks of stroke and CVD [1, 54].

The translated questionnaire was administered to all eligible people irrespective of their level of CVD risk. A possible limitation is that the reliability testing consisted only the Malay ethnic group, which was not representative of the multiracial Malaysian population. However, the translated questionnaire should be suitable to the Malaysians as this study considers the linguistics of the questionnaire and cross-cultural adaptation of Malay-spoken users regardless of ethnicity.

Future studies assessing populations at increased risk of CVD should consider including smoking and alcohol consumption to learn about these individuals’ knowledge, perception and/or behaviours towards the risk. Additional studies should be conducted with the involvement of larger samples from multiracial participants to confirm the generalisability of these findings.

## Conclusions

The translated and adapted questionnaire (ABCD-*M*) showed evidence of reliability and validity in assessing the awareness of stroke and CVD risk in the general population. The applicability of the questionnaire has been tested in a broad spectrum of Malaysian demography irrespective of different levels of CVD risk. The questionnaire effectively empowers population-wide to make informed lifestyle choices about their health and will provide some clues to the government in improving and strengthening the policies related to NCD prevention, especially for stroke and CVDs.

## Supplementary Information

**Additional file 1.**

**Additional file 2.**

**Additional file 3: Table 1.** Content validity index: the relevance ratings on the item scale by five experts. **Table 2.** Face validity index: the clarity and comprehension ratings on the item scale by 10 users.

## Data Availability

The authors confirm that the data supporting the findings of this study are available within the article. All data generated or analysed during this study are included in this published article [and its supplementary information files].
