# Supplementary material for: The Malay version of the attitudes and beliefs about cardiovascular disease (ABCD-M) risk questionnaire: a translation, reliability and validation study
Source: BMC Public Health. 2022 Jul 25;22:1412. doi: 10.1186/s12889-022-13811-8 (PMC9310389; doi:10.1186/s12889-022-13811-8)
Supplement: Supplementary file 2 — Additional file 2. [file 12889_2022_13811_MOESM2_ESM.pdf]

The ABCD Risk Questionnaire and Scoring guide
Soal Selidik ABCD-M dan Panduan Pemarkahan

Scale	Item	Answer
**1. Knowledge**<br>***Pengetahuan***<br><br>(8 items)<br><br>Higher sum score = More knowledgeable/ more correct about having a stroke	1. One of the main causes of heart attack and stroke is stress.<br>*Salah satu punca utama serangan jantung dan strok ialah stres.*<br><br>2. Walking and gardening are considered types of exercise that can lower the risk of having a heart attack or stroke.<br>*Berjalan dan berkebun dianggap jenis senaman yang boleh mengurangkan risiko mendapat serangan jantung atau strok.*<br><br>3. Moderately intense activity of 2 ½ hours a week will reduce your chances of having a heart attack or stroke.<br>*Aktiviti sederhana berat yang dilakukan selama 2 ½ jam seminggu akan mengurangkan kemungkinan anda untuk mendapat serangan jantung atau strok.*<br><br>4. People who have diabetes are at higher risk of having a heart attack or stroke.<br>*Orang yang menghidap kencing manis mempunyai risiko yang lebih tinggi mendapat serangan jantung atau strok.*<br><br>5. Managing your stress levels will help you to manage your blood pressure.<br>*Mengurus tahap stres anda akan dapat membantu anda menguruskan tekanan darah anda.*<br><br>6. Drinking high levels of alcohol can increase your cholesterol and triglyceride levels.<br>*Minum alkohol yang tinggi kandungannya boleh meningkatkan tahap kolesterol dan trigliserida anda.*<br><br>7. HDL refers to ‘good’ cholesterol, and LDL refers to ‘bad’ cholesterol.<br>*HDL merujuk kepada kolesterol ‘baik’, dan LDL merujuk kepada kolesterol ‘buruk’.*	Q1-T<br>Q2-T<br>Q3-T<br>Q4-T<br>Q5-T<br>Q6-T<br>Q7-T<br>Q8-F<br><br>T= True<br>F= False<br><br>Correct: Score = 1, Incorrect or Don’t Know: Score = 0.

	8. A family history of heart disease is not a risk factor for high blood pressure.<br>*Sejarah keluarga yang mempunyai penyakit jantung bukan faktor risiko untuk tekanan darah tinggi.*	
**2. Perceived CVD Risk**<br>***Persepsi Risiko Strok***<br><br>(8 items)<br><br>Higher sum score = higher perception of risk of having a stroke	9. I feel I will suffer from a heart attack or stroke sometime during my life.<br>*Saya rasa saya akan mengalami serangan jantung atau strok pada satu ketika semasa hidup saya.*<br><br>10. It is likely that I will suffer from a heart attack or stroke in the future.<br>*Ada kemungkinan saya akan mengalami serangan jantung atau strok pada masa hadapan.*<br><br>11. It is likely that I will have a heart attack or stroke some time during my life.<br>*Ada kemungkinan saya akan mengalami serangan jantung atau strok pada satu ketika semasa hidup saya.*<br><br>12. There is a good chance I will experience a heart attack or stroke in the next 10 years.<br>*Ada kemungkinan besar saya akan mengalami serangan jantung atau strok dalam 10 tahun akan datang.*<br><br>13. My chances of suffering from a heart attack or stroke in the next 10 years are great.<br>*Kemungkinan saya mengalami serangan jantung atau strok dalam 10 tahun akan datang adalah tinggi.*<br><br>14. It is likely I will have a heart attack or stroke because of my past and/or present behaviours.<br>*Ada kemungkinan saya akan mengalami serangan jantung atau strok kerana tingkah laku saya yang lalu dan/atau sekarang.*<br><br>15. I am not worried that I might have a heart attack or stroke.<br>*Saya tidak risau bahawa saya mungkin mengalami serangan jantung atau strok.*	**Q9 – 14 and 16;**<br><br>1=Strongly disagree; 2=disagree; 3=agree; 4=strongly agree; N/A = 0<br><br>**Q15 Reverse coded**<br><br>4=Strongly disagree; 3=disagree; 2=agree; 1=strongly agree; N/A = 0

	16. I am concerned about the likelihood of having a heart attack or stroke in the near future.<br>*Saya bimbang mengenai kemungkinan mendapat serangan jantung atau strok tidak lama lagi.*	
**3. Perceived Benefits**<br>***Persepsi Kebaikan***<br><br>(4 items)<br><br>Higher average score = Higher perceived benefits of diet and exercise	17. When I exercise for at least 2½ hours a week I am doing something good for the health of my heart.<br>*Apabila saya bersenam sekurang-kurangnya 2½ jam seminggu saya melakukan sesuatu yang baik untuk kesihatan jantung saya.*<br><br>18. I am confident that I can maintain a healthy weight by exercising at least 2½ hours a week within the next two months.<br>*Saya yakin saya dapat mengekalkan berat badan yang sihat dengan bersenam sekurang-kurangnya 2½ jam seminggu dalam tempoh dua bulan akan datang.*<br><br>19. When I eat at least five portions of fruit and vegetables a day I am doing something good for the health of my heart.<br>*Apabila saya makan sekurang-kurangnya lima hidangan buah-buahan dan sayur-sayuran sehari saya melakukan sesuatu yang baik untuk kesihatan jantung saya.*<br><br>20. Increasing my exercise to at least 2½ hours a week will decrease my chances of having a heart attack or stroke.<br>*Meningkatkan senaman saya untuk sekurang-kurangnya 2½ jam seminggu akan mengurangkan kemungkinan saya mendapat serangan jantung atau strok.*	**Q17 – 20;**<br><br>1=Strongly disagree; 2=disagree; 3=agree; 4=strongly agree; N/A = 0
**4. Intentions to Change**<br>***Niat untuk Berubah***<br><br>(6 items)<br><br>Higher average score = Higher perceived readiness for change with	21. I am thinking about exercising at least 2½ hours a week.<br>*Saya memikirkan untuk bersenam sekurang-kurangnya 2½ jam seminggu.*<br><br>22. I intend or want to exercise at least 2½ hours a week.<br>*Saya berhasrat atau mahu bersenam sekurang-kurangnya 2½ jam seminggu.*	**Q21, 22, 24 and 25;**<br><br>1=Strongly disagree; 2=disagree; 3=agree; 4=strongly agree; N/A = 0

regard to exercise behaviour and health dietary behaviour	23. I am not thinking about exercising for 2 ½ hours a week.<br>*Saya tidak memikirkan untuk bersenam selama 2 ½ jam seminggu.*<br><br>24. I am confident that I can eat at least five portions of fruit and vegetables per day within the next two months.<br>*Saya yakin saya boleh makan sekurang-kurangnya lima hidangan buah-buahan dan sayur-sayuran setiap hari dalam tempoh dua bulan akan datang.*<br><br>25. I am thinking about eating at least five portions of fruit and vegetables a day.<br>*Saya memikirkan untuk makan sekurang-kurangnya lima hidangan buah-buahan dan sayur-sayuran setiap hari.*<br><br>26. I am not thinking about eating at least five portions of fruit and vegetables a day.<br>*Saya tidak memikirkan untuk makan sekurang-kurangnya lima hidangan buah-buahan dan sayur-sayuran setiap hari.*	**Q23 and 26 Reverse coded**<br><br>4=Strongly disagree; 3=disagree; 2=agree; 1=strongly agree; N/A = 0
