# Supplementary material for: The Malay version of the attitudes and beliefs about cardiovascular disease (ABCD-M) risk questionnaire: a translation, reliability and validation study
Source: BMC Public Health. 2022 Jul 25;22:1412. doi: 10.1186/s12889-022-13811-8 (PMC9310389; doi:10.1186/s12889-022-13811-8)
Supplement: Supplementary file 3 — Additional file 3: Table 1. Content validity index: the relevance ratings on the item scale by five experts. Table 2. Face validity index: the clarity and comprehension ratings on the item scale by 10 users. [file 12889_2022_13811_MOESM3_ESM.docx]

**Supplementary material**

**Table 1. Content validity index: the relevance ratings on the item scale by five experts**

| **Item** | **E^a^1** | **E2** | **E3** | **E4** | **E5** | **I-CVI^b^** |
| --- | --- | --- | --- | --- | --- | --- |
| Q1 | 1 | 1 | 1 | 1 | 1 | 1 |
| Q2 | 1 | 1 | 1 | 1 | 1 | 1 |
| Q3 | 1 | 1 | 1 | 1 | 1 | 1 |
| Q4 | 1 | 1 | 1 | 1 | 1 | 1 |
| Q5 | 1 | 1 | 1 | 1 | 1 | 1 |
| Q6 | 1 | 1 | 1 | 1 | 1 | 1 |
| Q7 | 1 | 1 | 1 | 1 | 1 | 1 |
| Q8 | 0 | 1 | 1 | 1 | 1 | 0.80 |
| Q9 | 1 | 0 | 1 | 1 | 1 | 0.80 |
| Q10 | 1 | 1 | 1 | 1 | 1 | 1 |
| Q11 | 1 | 1 | 1 | 1 | 1 | 1 |
| Q12 | 1 | 0 | 1 | 1 | 1 | 0.80 |
| Q13 | 1 | 0 | 1 | 1 | 1 | 0.80 |
| Q14 | 1 | 1 | 1 | 1 | 1 | 1 |
| Q15 | 1 | 1 | 1 | 1 | 1 | 1 |
| Q16 | 1 | 1 | 1 | 1 | 1 | 1 |
| Q17 | 1 | 1 | 1 | 1 | 1 | 1 |
| Q18 | 1 | 1 | 1 | 1 | 1 | 1 |
| Q19 | 1 | 1 | 1 | 1 | 1 | 1 |
| Q20 | 1 | 1 | 1 | 1 | 1 | 1 |
| Q21 | 1 | 0 | 1 | 1 | 1 | 0.80 |
| Q22 | 1 | 1 | 1 | 1 | 1 | 1 |
| Q23 | 0 | 1 | 1 | 1 | 0 | 0.60 |
| Q24 | 1 | 1 | 1 | 1 | 1 | 1 |
| Q25 | 1 | 1 | 1 | 1 | 1 | 1 |
| Q26 | 1 | 1 | 1 | 1 | 0 | 0.80 |
| **Content validity index average** | | | | | | **0.94** |

Note: **a**=expert; **b**=item-level content validity index

**Table 2. Face validity index: the clarity and comprehension ratings on the item scale by 10 users**

| **Item** | **R^a^1** | **R2** | **R3** | **R4** | **R5** | **R6** | **R7** | **R8** | **R9** | **R10** | **I-FVI^b^** |
| --- | --- | --- | --- | --- | --- | --- | --- | --- | --- | --- | --- |
| Q1 | 1 | 1 | 1 | 1 | 1 | 1 | 1 | 1 | 1 | 1 | 1 |
| Q2 | 1 | 1 | 1 | 1 | 1 | 1 | 1 | 1 | 1 | 1 | 1 |
| Q3 | 1 | 1 | 1 | 1 | 1 | 1 | 1 | 1 | 1 | 1 | 1 |
| Q4 | 1 | 1 | 1 | 1 | 1 | 1 | 1 | 1 | 1 | 1 | 1 |
| Q5 | 1 | 1 | 1 | 1 | 1 | 1 | 1 | 1 | 1 | 1 | 1 |
| Q6 | 1 | 1 | 1 | 1 | 1 | 1 | 1 | 1 | 1 | 1 | 1 |
| Q7 | 1 | 1 | 1 | 1 | 1 | 1 | 1 | 1 | 1 | 1 | 1 |
| Q8 | 1 | 1 | 1 | 1 | 1 | 1 | 1 | 1 | 1 | 1 | 1 |
| Q9 | 1 | 1 | 1 | 1 | 1 | 1 | 1 | 0 | 1 | 1 | 0.9 |
| Q10 | 1 | 1 | 1 | 1 | 1 | 1 | 1 | 1 | 1 | 1 | 1 |
| Q11 | 1 | 1 | 1 | 1 | 1 | 1 | 1 | 1 | 1 | 1 | 1 |
| Q12 | 1 | 1 | 1 | 1 | 1 | 1 | 1 | 1 | 1 | 1 | 1 |
| Q13 | 1 | 1 | 1 | 1 | 1 | 1 | 1 | 1 | 1 | 1 | 1 |
| Q14 | 1 | 1 | 1 | 1 | 1 | 1 | 1 | 1 | 1 | 1 | 1 |
| Q15 | 1 | 1 | 1 | 1 | 1 | 1 | 1 | 1 | 1 | 1 | 1 |
| Q16 | 1 | 1 | 1 | 1 | 1 | 1 | 1 | 1 | 1 | 1 | 1 |
| Q17 | 1 | 1 | 1 | 1 | 1 | 1 | 1 | 1 | 1 | 1 | 1 |
| Q18 | 1 | 1 | 1 | 1 | 1 | 1 | 1 | 1 | 1 | 1 | 1 |
| Q19 | 1 | 1 | 1 | 1 | 1 | 1 | 1 | 1 | 1 | 1 | 1 |
| Q20 | 1 | 1 | 1 | 1 | 1 | 1 | 1 | 1 | 1 | 1 | 1 |
| Q21 | 1 | 1 | 1 | 1 | 1 | 1 | 1 | 1 | 1 | 1 | 1 |
| Q22 | 1 | 1 | 1 | 1 | 1 | 1 | 1 | 1 | 1 | 1 | 1 |
| Q23 | 1 | 1 | 1 | 1 | 1 | 1 | 1 | 1 | 1 | 1 | 1 |
| Q24 | 1 | 1 | 1 | 1 | 1 | 1 | 1 | 1 | 1 | 1 | 1 |
| Q25 | 1 | 1 | 1 | 1 | 1 | 1 | 1 | 1 | 1 | 1 | 1 |
| Q26 | 1 | 1 | 1 | 1 | 1 | 1 | 1 | 1 | 1 | 1 | 1 |
| **Face validity index average** | | | | | | | | | | | **0.99** |

Note: **a**=respondent; **b**=item-level face validity index
